# Hepatitis C screening in Lithuania: first-year results and scenarios for achieving WHO elimination targets

**DOI:** 10.1186/s12889-024-18470-5

**Published:** 2024-04-15

**Authors:** Janina Petkevičienė, Alexis Voeller, Eglė Čiupkevičienė, Devin Razavi-Shearer, Valentina Liakina, Ligita Jančorienė, Edita Kazėnaitė, Viačeslavas Zaksas, Gediminas Urbonas, Limas Kupčinskas

**Affiliations:** 1 Health Research Institute, Faculty of Public Health, Lithuanian University of Health Sciences, Tilžės str. 18, LT47181, Kaunas, Lithuania; 2 Department of Preventive Medicine, Faculty of Public Health, Lithuanian University of Health Sciences, Tilžės str. 18, LT47181, Kaunas, Lithuania; 3 Center for Disease Analysis Foundation, 1120 W South Boulder Rd, Suite 102, Lafayette, CO USA; 4 Faculty of Medicine, Vilnius University, Universiteto str. 3, LT01513, Vilnius, Lithuania; 5 Faculty of Fundamental Sciences, Vilnius Tech, Saulėtekio av. 11, LT10223, Vilnius, Lithuania; 6 Clinic of Infectious Diseases and Dermatovenerology, Institute of Clinical Medicine, Medical Faculty, Vilnius University, Santariškių str. 14, 08406 Vilnius, Lithuania; 7 Vilnius University Hospital Santaros Klinikos, Santariškių str. 2, LT08661, Vilnius, Lithuania; 8 National Health Insurance Fund under the Ministry of Health, Europos Sq. 1, LT03505, Vilnius, Lithuania; 9 Department of Family Medicine, Lithuanian University of Health Sciences, Eivenių str. 2, LT50161, Kaunas, Lithuania; 10 Department of Gastroenterology, Lithuanian University of Health Sciences, Eivenių str. 2, LT50161, Kaunas, Lithuania

**Keywords:** Hepatitis C, Screening, Elimination, World Health Organisation targets, Mathematical modelling, Lithuania

## Abstract

**Background:**

The World Health Organization (WHO) has outlined a set of targets to achieve eliminating hepatitis C by 2030. In May 2022, Lithuanian health authorities initiated a hepatitis C virus (HCV) screening program to start working towards elimination. In the program, bonus was given to general practitioners (GPs) to promote and conduct anti-HCV tests for two situations: (1) one time testing for individuals born in 1945–1994 and (2) annual HCV testing for persons who inject drugs or are living with human immunodeficiency virus (HIV) regardless of age. This study aimed to model the current viral hepatitis C epidemiological status in Lithuania and to outline the requirements for WHO elimination targets using the first-year HCV screening results.

**Methods:**

Individuals were invited to participate in the anti-HCV screening by GPs during routine visits. Patients who tested positive were then referred to a gastroenterologist or infectious disease doctor for further confirmatory testing. If a patient received a positive RNA test and a fibrosis staging result of ≥ F2, the doctor prescribed direct-acting antivirals. Information on the patients screened, diagnosed, and treated was obtained from the National Health Insurance Fund. The Markov disease progression model, developed by the CDA Foundation, was used to evaluate the screening program results and HCV elimination progress in Lithuania.

**Results:**

Between May 2022 and April 2023, 790,070 individuals underwent anti-HCV testing, with 11,943 individuals (1.5%) receiving positive results. Anti-HCV seroprevalence was found to be higher among males than females, 1.9% and 1.2%, respectively. Within the risk population tested, 2087 (31.1%) seropositive individuals were identified. When comparing the screening program results to WHO elimination targets through modelling, 2180 patients still need to be treated annually until 2030, along with expanding fibrosis restrictions. If an elimination approach was implemented, 1000 new infections would be prevented, while saving 150 lives and averting 90 decompensated cirrhosis cases and 110 hepatocellular carcinoma cases.

**Conclusions:**

During the first year of the Lithuanian screening program, GPs were able to screen 44% of the target population. However, the country will not meet elimination targets as it currently stands without increasing treatment levels and lifting fibrosis restrictions.

## Background

Viral hepatitis C (HCV) infections are a significant global public health challenge, affecting approximately 1% of the world’s population [1]. Currently, there is no vaccine to prevent the transmission of HCV. An estimated 5 million Europeans are chronically infected, with prevalence rates of HCV infection varying significantly among different European Union countries [2]. Furthermore, chronic hepatitis C is one of the leading contributors to liver disease-related death due to serious complications, such as liver cirrhosis and hepatocellular carcinoma [3, 4].

In 2016, the World Health Organization (WHO) set an ambitious goal to eliminate hepatitis C as a public health threat by 2030, targeting an 80% reduction in new chronic infections, a 65% reduction in mortality associated with HCV infection, an increase to 90% of diagnosed and 80% of patients treated in comparison to 2015 data [5]. With the introduction of direct-acting antivirals (DAAs), a comprehensive treatment regimen, the WHO elimination targets are achievable. These medications demonstrate high sustained virologic response rates (SVR about 98%), have fewer side effects, and offer simplified regimens compared to interferon-based therapies [6, 7]. With DAAs, patients can initiate treatment at earlier disease stages, which allows for the potential to improve clinical outcomes and minimize viral transmission. When HCV patients initiate treatment earlier in the course of the disease, some studies have demonstrated its cost-effectiveness compared to delaying treatment initiation until the disease reaches more advanced stages [8, 9]. However, globally, a significant number of infected individuals remain undiagnosed, placing them at high risk of developing cirrhosis and hepatocellular carcinoma. It has been estimated that only 36% of Europeans with viraemic HCV infection had received a diagnosis [10]. Underdiagnosis of HCV patients highlights the crucial role screening programs play in identifying asymptomatic infected individuals before advanced complications may arise.

When implementing a screening program, countries have the option to adopt various strategies, such as universal screening of the entire population or targeted screening for specific populations considered to be at risk for contracting HCV. There is a continuing debate as to which of these strategies is more cost-effective [11–14]. Most European countries have implemented targeted screenings, in hopes to move towards achieving HCV elimination by 2030 [2]. Meanwhile, Iceland and Georgia have adopted a mass screening approach to diagnose and treat HCV infected individuals within their countries [15, 16].

Reliable epidemiological data are essential to support the development of a national screening strategy. At the conception of the national screening program, Lithuania did not have sufficient representative data for the prevalence of HCV infection to fully understand the epidemiological situation within the country. The first attempt to assess the prevalence of anti-HCV in the Lithuanian population was made in 2011 when a study outlined a prevalence of 2.78% in 1528 adults from 5 cities of the country [17]. To expand upon this effort, in 2020–2022, a pilot study was carried out in a primary healthcare centre in Klaipėda, aiming to assess the seroprevalence of HCV antibodies (anti-HCV) and to evaluate the possibility of a HCV screening program in a primary healthcare setting in Lithuania [18]. After screening 4867 individuals, an anti-HCV seroprevalence of 1.7% was observed. The majority (97.5%) of identified anti-HCV-positive cases occurred among adults born between 1945 and 1994. Individuals who underwent blood transfusions or donated blood before 1993, those with tattoos, illicit injection drug users, and former prisoners showed higher anti-HCV seroprevalence. This study helped demonstrate how individuals would participate in a screening program conducted by GPs in similar settings across the country [18]. This framework further became the basis for creating a national screening program.

National HCV screening started on May 5, 2022, when the order of the Minister of Health was issued. A special bonus was given to GPs to promote and conduct anti-HCV serological tests for two specific groups: (1) once in the lifetime testing for individuals born between 1945 and 1994 and (2) annual HCV testing for the risk group, which includes persons who inject drugs (PWID) or individuals living with human immunodeficiency virus (HIV), regardless of age. Such an initiative was one of the first in Central and Eastern Europe.

All primary healthcare centres throughout the country participated in the screening. Individuals were invited to be screened during their routine visits with their GPs. The screening involved a serum blood test to detect the presence of HCV antibodies. Enzyme-linked immunosorbent assay (ELISA) was used to detect HCV antibodies from a blood serum test. For providers to collect their bonus (14.30 Euros in 2022 and 15.44 Euros in 2023 for every tested person), information on the number of tests performed was included in an approved statistical form, which was submitted to the National Health Insurance Fund.

Individuals who received positive test results were subsequently referred to either a gastroenterologist or an infectious disease doctor, where the patients received confirmatory HCV ribonucleic acid (RNA) testing. If a positive RNA result was received, patients underwent liver fibrosis staging using transient elastography (FibroScan). For treatment, according to the existing guidelines, the doctor can prescribe DAA treatment if the determined liver fibrosis stage is F2 or higher. In this program, diagnostic and treatment services were provided free of charge.

With the data collected from the screening program, this study aimed to model the current viral hepatitis C epidemiological status in Lithuania and to outline the required interventions needed to reach WHO elimination targets.

## Methods

### HCV screening data

Data aggregated by sex and ten-year age groups regarding screened and seropositive individuals in the birth cohort and risk group (PWID or individuals living with HIV), were received from the National Health Insurance Fund. Anti-HCV seroprevalence was assessed as the number of individuals with positive test results divided by the number of all screened individuals. The proportion of anti-HCV seropositive individuals in different sex and age groups was compared using a χ^2^ test, Z-test with Bonferroni correction for multiple comparisons and a Fisher Exact test. Data analysis was performed using the statistical package IBM SPSS Statistics for Windows, Version 27.0 (IBM Corp.: Armonk, NY, USA, released 2020).

### Disease burden model

A previously published Markov disease progression model in Microsoft Excel® (Microsoft Corporation) was utilized to evaluate the number of HCV infections within the country with the application of Lithuania-specific epidemiological inputs [19]. The model followed HCV disease progression from acute infection to chronic infection through end-stage liver disease, liver-related mortality, background mortality, or eventual cure. For each disease stage, the model estimated the number of annual new (incident) cases by taking the product of the prevalent population partitioned by sex and one-year age group by annual progression rates from the earlier disease state. The model also considered all-cause mortality based on Lithuania-specific demographic data. The population data, mortality rates, and birth rates for Lithuania were obtained from the United Nations World Population Prospects 2022 database, with the data being separated by sex and five-year age groups from 1950 to 2030 [20]. A probabilistic sensitivity analysis (PSA) was used to generate 95% uncertainty intervals (UIs) on modelled outputs with Crystal Ball (version 11.1.2.3.500), an Excel add-in by Oracle, with 1000 Monte Carlo simulations to account for the assumed uncertain value within this study: inputted prevalence. It was assumed that the prevalence had a beta-PERT distribution.

### Epidemiological inputs

The country-specific inputs included viraemic prevalence by age and sex, treatment, and diagnosis data (Table 1). These data were originally collected through the Polaris Observatory through annual surveys. With the initiation of the HCV screening program, input data was updated with the screening results. The screening program data provided the number of anti-HCV cases since the beginning of the program, along with the viraemic rate. These data were further adjusted to represent the Lithuanian population and further modelled to estimate the number of HCV RNA-positive cases within the country starting in 1950 through 2030.

From the HCV screening program, an anti-HCV prevalence estimate of 1.51% was obtained and applied to the entire population in the country to calculate the population-weighted prevalence. Viraemic prevalence by age and sex was also calculated by adjusting the screening program’s anti-HCV data for the total population. The annual number of treated patients was taken from the database of the National Health Insurance Fund [21]. Liver transplant data were obtained from the database of the Lithuanian National Transplant Bureau [22].

**Table 1:** HCV disease burden model input parameters

Parameter	Input	Year of estimate	Source
Anti-HCV prevalence	1.51%	2022–2023	First-year screening data
Anti-HCV prevalence low	1.20%	2022–2023	First-year screening data
Anti-HCV prevalence high	1.90%	2022–2023	First-year screening data
Newly viraemic diagnosed	11,769	2022–2023	Calculated from screening data
Number treated	5269	2022–2023	National Health Insurance Fund [21]
Viraemic rate	58.2%	2022–2023	Calculated from the data of pilot study in Klaipeda [18]
Liver transplants	32	2022	Lithuanian National Transplant Bureau [22]

### Scenarios

Three different scenarios were created within the model to estimate the HCV disease burden in Lithuania before and after the implementation of the HCV screening program. Within these scenarios, the burden of hepatitis within the country was examined through end-stage liver disease outcomes: HCV-related deaths, cases of hepatocellular carcinoma and cases of decompensated cirrhosis. The scenarios used within the analysis are described below.

1. Standard of Care prior to 2022

This scenario looked at the status quo within Lithuania before the implementation of any HCV elimination programs. Treatment and diagnosis levels from this timeline are outlined in Table 2. This scenario assumed that fibrosis restrictions were in place only allowing patients F2 and higher access to treatment, along with restricting treatment to patients aged 15–74.

2. National Screening Program

This scenario takes considered the implementation of the HCV screening program starting on May 5, 2022. The data from this program suggests an anti-HCV prevalence of 1.51% among tested individuals. Treatment and diagnosis levels starting in 2022 are outlined in Table 2. This scenario assumed that fibrosis restrictions were in place by only allowing patients F2 and higher access to treatment, along with restricting treatment to patients aged 15–74.

3. WHO Elimination

This scenario achieved HCV elimination targets set by the WHO in Lithuania by 2030. Elimination required increasing interventions within the country to achieve an 80% reduction in new infections, a 65% reduction in liver-related deaths from 2015 by 2030, 90% diagnosis coverage, and 80% of those diagnosed treated. All fibrosis restrictions were lifted within this scenario, and ages up to 84 were eligible for treatment.

**Table 2:** Disease input parameters for the scenarios

Scenarios	2020	2022	2023	2024	2025	2030
**Treated**						
Standard of Care prior to 2022	930	960	960	960	960	960
Screening Program	930	1570	3700	960	960	960
WHO Elimination	930	1570	3700	2180	2180	2180
**Viraemic newly diagnosed**						
Standard of Care prior to 2022	1520	1920	1920	1920	1920	1920
Screening Program	930	4360	7410	960	960	960
WHO Elimination	930	4360	7410	500	500	500
**Liver fibrosis stage for treatment eligibility**,						
Standard of Care prior to 2022	≥F2	≥F2	≥F2	≥F2	≥F2	≥F2
Screening Program	≥F2	≥F2	≥F2	≥F2	≥F2	≥F2
WHO Elimination	≥F2	≥F2	≥F2	≥F0	≥F0	≥F0
**Anti-HCV tests**						
Screening Program		460,250	851,380			
WHO Elimination		460,250	851,380	488,370	740	270
Incident cases of chronic HCV*						
Screening Program	610	580	570	560	550	500
WHO Elimination	610	580	570	520	450	160
Age limits for treatment eligibility*						
Screening Program	15–74	15–74	15–74	15–74	15–74	15–74
WHO Elimination	15–74	15–74	15–74	15–84	15–84	15–84
Sustained virological response	99%	99%	99%	99%	99%	99%

*Modelled outputs

## Results

### Screening program

At the beginning of 2022, approximately 1.8 million individuals born between 1945 and 1994 resided in Lithuania. Between May 5, 2022 and April 30, 2023, a total of 790,070 individuals underwent HCV antibody testing (Table 3), with 41.8% being males and 58.2% females. This resulted in the screening of 44% of the target population during the first year of the program. Most of the subjects examined (783,375) belonged to the birth cohort of 1945–1994. Additionally, 6695 individuals were screened who were considered to be part of the risk group.

**Table 3:** Number of screened individuals in 1945–1994 birth cohort and risk group by age and sex

Sex	Year of birth							Total
	1935–1944	1945–1954	1955–1964	1965–1974	1975–1984	1985–1994	> 1994	
	**1945–1994 birth cohort**							
Males	-	49,286	85,774	82,995	66,095	42,969	-	327,119
Females	-	87,167	125,967	109,345	69,689	64,088	-	456,256
Total	-	136,453	211,741	192,340	135,784	107,057	-	783,375
	**Risk group**							
Males	28	447	732	853	748	488	51	3347
Females	59	728	924	767	420	395	55	3348
Total	87	1175	1656	1620	1168	883	106	6695
	**1945–1994 birth cohort and risk group**							
Males	28	49,733	86,506	83,848	66,843	43,457	51	330,466
Females	59	87,895	126,891	110,112	70,109	64,483	55	459,604
Total	87	137,628	213,397	193,960	136,952	107,940	106	790,070

Positive anti-HCV test results were found in 11,943 (1.5%) individuals (Table 4). Anti-HCV seroprevalence was higher among males than females, 1.9% and 1.2%, respectively. The difference was noticeable across all age groups except for the eldest individuals born between 1945 and 1954. Among males born between 1965 and 1984, the highest anti-HCV seroprevalence was identified. The lowest seroprevalence of anti-HCV was among women born between 1985 and 1994.

Within the risk group, 2087 seropositive PWID and individuals living with HIV were identified, demonstrating an almost 30-fold higher anti-HCV seroprevalence compared to the 1945–1994 birth cohort, 31.1% and 1.3%, respectively (Table 4). Across all age groups, no significant differences between males and females were observed, except among the youngest individuals born after 1994. In this age group, the prevalence among males was 21.6%, while among females, it was 5.5%.

**Table 4:** Anti-HCV seroprevalence in 1945–1994 birth cohort and risk group by age and sex

Sex	Year of birth							Total
	1935–1944	1945–1954	1955–1964	1965–1974	1975–1984	1985–1994	> 1994	
	**1945–1994 birth cohort**							
Males N (%)	-	588 (1.2)	1334 (1.6*)	1590 (1.9*^#^)	1220 (1.8*^#^)	491 (1.1*)	-	5223 (1.6*)
Females N (%)	-	946 (1.1)	1419 (1.1)	1219 (1.1)	675 (1.0)	374 (0.6^#^)	-	4633 (1.0)
Total N (%)	-	1534 (1.1)	2753 (1.3)	2809 (1.5^#^)	1895 (1.4)	865 (0.8)	-	9856 (1.3)
	**Risk group**							
Males N (%)	2 (7.1)	117 (26.2)	256 (35.0)	300 (35.2)	250 (33.4)	147 (30.1)	11 (21.6**)	1083 (32.3)
Females N (%)	4 (6.8)	159 (21.8)	288 (31.2)	248 (32.3)	149 (35.5)	153 (38.7)	3 (5.5)	1004 (29.9)
Total N (%)	6 (6.9)	276 (23.5)	544 (32.9)	548 (33.8)	399 (34.2)	300 (34.0)	14 (13.2)	2087 (31.1)
	**1945–1994 birth cohort and risk group**							
Males N (%)	2 (7.1)	705 (1.4)	1590 (1.8*)	1890 (2.3*^#^)	1470 (2.2*^#^)	638 (1.5*)	11 (21.6*)	6306 (1.9*)
Females N (%)	4 (6.8)	1105 (1.3)	1707 (1.3)	1467 (1.3)	824 (1.2)	527 (0.8)	3 (5.5)	5637 (1.2)
Total N (%)	6 (6.9)	1810 (1.3)	3297 (1.5)	3357 (1.7^#^)	2294 (1.7^#^)	1165 (1.1)	14 (13.2)	11,943 (1.5)

* *P* < 0.001 compared with females; ** *P* = 0.02 compared with females; ^#^*P* < 0.001 compared with other age groups

The screening coverage correlated with the respective sizes of the counties (Table 5). The seroprevalence of anti-HCV ranged from 1.2 to 1.9% across ten counties, with the highest prevalence observed in Klaipėda county.

**Table 5:** Number of screened individuals and anti-HCV seroprevalence in counties of Lithuania

County	Number of screened individuals	Number of anti-HCV seropositive individuals	Seroprevalence of anti-HCV (%)
Alytus	25,708	370	1.4
Kaunas	182,645	2745	1.5
Klaipėda	94,638	1792	1.9*
Marijampolė	37,344	538	1.4
Panevėžys	59,904	732	1.2
Šiauliai	88,723	1277	1.4
Tauragė	30,083	515	1.7
Telšiai	37,422	460	1.2
Utena	27,536	444	1.6
Vilnius	205,145	3027	1.5

*p < 0.001 compared to other counties

During the first year of HCV screening, 2581 patients received treatment with DAAs. The annual number of treated patients was more than 2-fold higher than observed in preceding years as a result of a larger diagnosis rate (Table 2).

### Disease burden modelling scenarios

With the model taking into consideration the various inputs observed within the screening program, an analysis comparing different scenarios was able to be carried out.

The Standard of Care prior to 2022 scenario estimated that there would be 21,400 infections, with only an estimated 43% (9300) being diagnosed through 2030. Of the total number of infections, only an estimated 7% (960) would be treated. In 2021, an estimated 90 liver-related deaths would have occurred, along with an estimated 70 hepatocellular carcinoma cases.

**Fig. 1:**
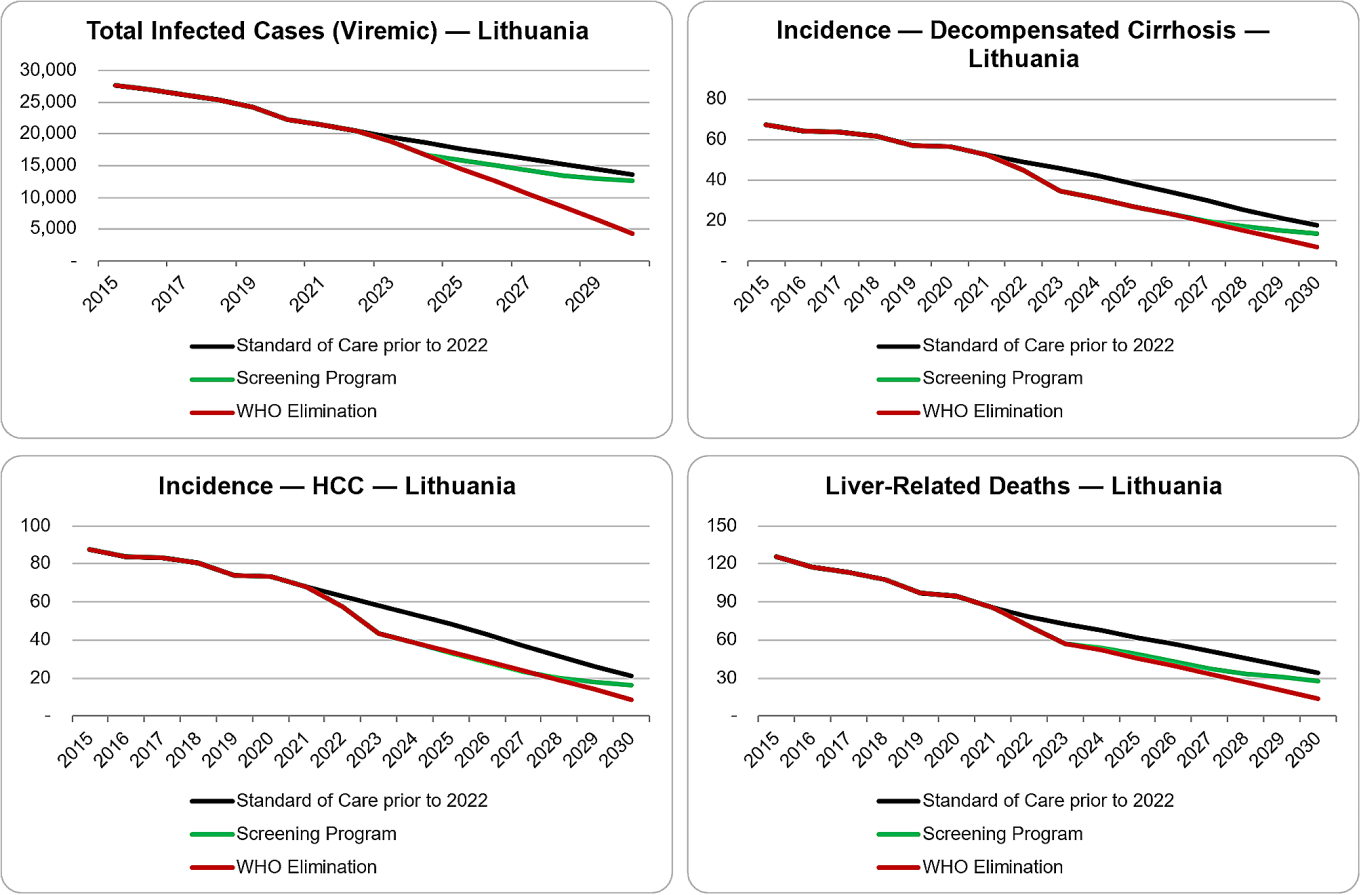
Differences between scenario outcomes. Hepatitis disease burden outcomes by scenario in Lithuania, modelled from 2015?2030: **(a)** Total number of viraemic HCV infections; **(b)** Total number of incident cases of decompensated cirrhosis; **(c)** Total number of incident cases of hepatocellular carcinoma; **(d)** Total number of incident cases of HCV liver-related deaths

The implementation of the National Screening Program increased the number of screens to 460,250 in 2022 and 851,380 in 2023 (Table 2). With the increase in screening, Lithuania diagnosed 4360 patients in 2022 and 7410 patients in 2023, along with treating 1570 patients in 2022 and 3700 patients in 2023 (Table 2). With these assumptions, is the model estimated that 20,400 (95% UI: 17,580–23,820) viraemic infections remained in the country at the beginning of 2022, decreasing to 18,900 (95% UI: 16,070–22,250) at the beginning of 2023. The HCV screening program also accounts for 61% (12,500) diagnosed through 2022, increasing to 96% (18,100) diagnosed through 2023. A similar pattern was estimated with treatment levels. Through 2022, an estimated 8% (1600) of the infected population was treated, increasing to 13% (2400) through 2023. With the screening program unchanging after 2023, by 2030, an estimated 12,600 viraemic infections will remain, with 98% (12,300) of the patients being diagnosed and 4% (460) of the total infections being treated. Compared to the Standard of Care prior to 2022 scenario, cumulative outcomes from 2015 to 2030 would result in 1 new infection avoided, 100 lives saved from liver-related deaths, 80 new decompensated cirrhosis cases averted, and 100 new hepatocellular carcinoma cases averted due to the introduction of an intervention program (Fig. 1).

WHO elimination targets were reached in Lithuania with an excess of 491,760 screens by 2030, with 488,370 (95% UI: 257,820–554,740) happening in 2024 (Table 2). There would also need to be 2180 patients treated annually starting in 2024 through 2030, with 500 patients diagnosed within the same time frame (Table 2). To reach elimination targets, the treatment eligibility in terms of fibrosis stage would have to be expanded to include F0 and F1 patients (Table 2). If all these things are implemented, an estimated 4380 (95% UI: 2010–7190) infections will remain at the beginning of 2030. Compared to the Standard of Care prior to 2022 scenario, cumulative outcomes from 2015 to 2030 resulted in 1000 new infections avoided, 150 lives saved from liver-related deaths, 90 new decompensated cirrhosis cases averted, and 110 new hepatocellular carcinoma cases averted (Fig. 1).

## Discussion

Effective HCV screening programs, enhanced treatment coverage with DAAs, and prevention strategies are crucial for achieving WHO HCV elimination targets by 2030 and addressing the challenges posed by HCV in Lithuania. This study analysed the results of the HCV screening program and further used them as inputs for estimating the disease burden in Lithuania and developing potential scenarios to achieve WHO elimination targets.

Our study revealed an active participation in HCV screening. The identified anti-HCV seroprevalence of 1.5% was similar to the prevalence in the neighbouring Baltic countries, with Estonia reporting 1.5–2.0% [23] and Latvia reporting 2.4% [24]. Additionally, comparable numbers of seropositive individuals were found in other Central European countries [25]. The seroprevalence of anti-HCV was consistent across all Lithuanian counties, ranging from 1.2 to 1.9%, with the highest prevalence observed in Klaipėda county. These findings align with our prior pilot study conducted in a single primary healthcare centre in Klaipėda city [18].

We observed a significantly higher seroprevalence of anti-HCV (31.1%) among PWID and individuals living with HIV, which is in line with the findings of other studies [26, 27]. Furthermore, an examination of data from a national surveillance system confirmed that injection drug use is the most frequently documented transmission route for acute HCV cases in Lithuania [28]. Historically, the HCV transmission routes have undergone notable changes in many countries [26, 27, 29]. Before screening assays became available, most HCV infections were iatrogenic, resulting from transfusions with infected blood or unsafe invasive medical and surgical procedures. A significant proportion of the older HCV-infected population in Lithuania likely acquired the infection through blood transfusions and blood donation before 1993 [18]. The majority of new HCV infections occur among PWID, and elimination strategies require particular focus on this population. Consequently, the decision to include annual testing for injection drug users in the Lithuanian screening program appears to be a reasonable strategy. In Lithuania, large outpatient clinics have mental health centres that provide health care services to PWID. They also refer PWID for anti-HCV testing, thereby increasing the likelihood of identifying those who are infected. PWID are a key risk group for HCV transmission in this country. There are several highly effective harm reduction interventions to prevent HCV transmission, such as opiate substitution therapies and high coverage needle and syringe programs [30, 31]. However, HCV prevention interventions for PWID remain non-existent in most countries, including Lithuania, and are likely to be insufficient to prevent HCV transmission [32]. The detection and treatment of HCV, along with the implementation of harm reduction strategies, are necessary for achieving WHO elimination goals.

Our data revealed that only the third scenario, characterized by the completion of mass screening by 2024, expanded treatment eligibility regardless of fibrosis stage and a sufficient number of treated patients, would allow Lithuania to achieve WHO elimination targets. In the coming month in 2024, Lithuania is expected to remove treatment restrictions on the fibrosis staging. Currently, treatment delays for HCV patients are attributed to prolonged waiting times for gastroenterologist or infectious disease doctor consultations and testing for HCV viremia. Furthermore, the substantial increase in the number of patients requiring treatment has led to a shortage of DAAs in the country, prompting health authorities to reassess contracts with pharmaceutical suppliers. Thus, organizational efforts are required to accelerate the process and remove barriers to linkage to care and treatment delivery [33].

Discussions are still ongoing regarding the optimal HCV screening strategies, considering factors such as effectiveness, costs, and access. Numerous studies have evaluated potential HCV screening and management strategies, revealing that multiple screening approaches and treatment with DAAs can be effectively implemented across diverse populations, demonstrating cost-effectiveness [11–14, 34–36]. Universal screening is recommended in countries with high HCV prevalence. Only a limited number of countries have adopted this screening approach, with Egypt as an example, where over 50 million people have undergone HCV screening and several million have received treatment [36]. In Iceland, a nationwide program for the treatment of all patients infected with HCV was launched, providing universal access to DAA. The focus was on identifying and treating individuals at high risk of transmitting HCV, specifically PWID and prison inmates. Additionally, harm reduction initiatives, such as the distribution of sterile needles and syringes, were implemented [15]. In 2015, Georgia, having an anti-HCV prevalence of 5.4%, launched a national HCV elimination program. The program introduced comprehensive testing, treatment, and prevention measures targeting the general population and PWID. Over the time period of six years, the HCV elimination program has demonstrated remarkable cost-effectiveness, successfully reducing prevalence and incidence by more than half [16].

Effective HCV screening strategies play a crucial role in global initiatives for early detection, treatment, and preventing the spread of HCV. It is essential to tailor these strategies to specific populations to optimize the impact of screening programs. Further country-specific studies are needed to assess the cost-effectiveness of HCV screening programs and provide evidence for informed policy decisions aimed at achieving HCV elimination.

This study has several limitations that need to be acknowledged. The screening program lacks an information system that would allow tracking of an individual from the anti-HCV testing to treatment outcomes. Different sources of information from primary health centres, laboratories and hospitals are difficult to merge.

The modelling used within this study also carries limitations that are innate in mathematical modelling and have been previously described [1, 19]. The greatest limitation within modelling is the availability and quality of data used which can greatly impact the outputs of the model. As prevalence is the only uncertain value assumed within this study, the use of uncertainty intervals can help address the weakness it creates. The use of empirical national data also helps minimize its effects. Yet, this still may not capture all the sources of bias modelling is prone too, including selection bias, sampling bias and measurement bias. This model further does not take into consideration coinfections or comorbidities.

## Conclusions

This study reveals an active engagement of individuals registered with primary healthcare centres in HCV screening conducted by their GPs. The screening strategy endorsed by Lithuanian health authorities shows potential for identification of the majority of HCV-infected individuals in the country. However, it is crucial to increase the number of patients undergoing treatment. Scenario modelling confirms that expanded treatment eligibility irrespective of fibrosis stage and increased treatment would facilitate the achievement of WHO HCV elimination targets in Lithuania by 2030.

## Data Availability

The data that support the findings of this study are available from the National Health Insurance Fund under the Ministry of Health (Lithuania), but restrictions apply to the availability of these data, which are not publicly available. Aggregated data are, however, available from the authors upon reasonable request and with permission of the National Health Insurance Fund.

## Abbreviations

Anti-HCV: HCV antibodies
CDA: Center for Disease Analysis
DAAs: Direct-acting antivirals
GP: General practitioner
HCV: Hepatitis C virus
HIV: Human immunodeficiency virus
PWID: Persons who inject drugs
RNA: Ribonucleic acid
WHO: World Health Organisation
